# Microfluidic-Based Multi-Organ Platforms for Drug Discovery

**DOI:** 10.3390/mi7090162

**Published:** 2016-09-08

**Authors:** Ahmad Rezaei Kolahchi, Nima Khadem Mohtaram, Hassan Pezeshgi Modarres, Mohammad Hossein Mohammadi, Armin Geraili, Parya Jafari, Mohsen Akbari, Amir Sanati-Nezhad

**Affiliations:** 1 BioMEMS and Bioinspired Microfluidic Laboratory, Department of Mechanical and Manufacturing Engineering, University of Calgary, 2500 University Drive NW, Calgary, AB T2N 1N4, Canada; ahmad.rezaei@polymtl.ca (A.R.K.); hassan.pezeshki@gmail.com (H.P.M.); 2 Laboratory for Innovations in MicroEngineering (LiME), Department of Mechanical Engineering, University of Victoria, Victoria, BC V8P 5C2, Canada; nkhadem@uvic.ca (N.K.M.); makbari@uvic.ca (M.A.); 3 Division of Medical Sciences, University of Victoria, Victoria, BC V8P 5C2, Canada; 4 Department of Biomedical Engineering, University of Wisconsin-Madison, Madison, WI 53706, USA; 5 Department of Chemical and Petroleum Engineering, Sharif University of Technology, Azadi Ave., Tehran 11155-9516, Iran; maosein@gmail.com (M.H.M.); armin.geraili@gmail.com (A.G.); 6 Department of Electrical Engineering, Sharif University of Technology, Azadi Ave., Tehran 11155-9516, Iran; parya74jafari@gmail.com; 7 Center for Bioengineering Research and Education, Biomedical Engineering Program, University of Calgary, 2500 University Drive NW, Calgary, AB T2N 1N4, Canada

**Keywords:** drug discovery, body-on-chip, microfluidics, organ-on-chip, in silico modeling

## Abstract

Development of predictive multi-organ models before implementing costly clinical trials is central for screening the toxicity, efficacy, and side effects of new therapeutic agents. Despite significant efforts that have been recently made to develop biomimetic in vitro tissue models, the clinical application of such platforms is still far from reality. Recent advances in physiologically-based pharmacokinetic and pharmacodynamic (PBPK-PD) modeling, micro- and nanotechnology, and in silico modeling have enabled single- and multi-organ platforms for investigation of new chemical agents and tissue-tissue interactions. This review provides an overview of the principles of designing microfluidic-based organ-on-chip models for drug testing and highlights current state-of-the-art in developing predictive multi-organ models for studying the cross-talk of interconnected organs. We further discuss the challenges associated with establishing a predictive body-on-chip (BOC) model such as the scaling, cell types, the common medium, and principles of the study design for characterizing the interaction of drugs with multiple targets.

## 1. Introduction

Metabolism and toxicity analysis is one of the essential steps for drug design [1,2]. Animal models have long been used for drug testing [1,3,4]. However, in vivo tests with animal models have a limited success when translating results to humans, as they are expensive and time-consuming, and are subjected to ethical considerations. In vitro and in silico models instead have been developed to overcome such limitations [5,6]. In vitro models are traditionally based on static cell cultures in plates and provide a simple interface for testing the metabolism and toxicity of candidate drugs [7,8,9]. However, these models lack the ability to mimic the in vivo cell-cell and cell-matrix interactions within the tissue microenvironment. Organoid-based culture models have significantly improved the standards of in vitro culture models but their application for the systemic analysis of the crosstalk of multiple organs is limited [10,11,12]. Microtechnology has significantly contributed to the development of biomimetic in vitro models for predicting the drug efficacy with higher reliability than traditional models and organoid systems [13,14,15]. This technology has also been utilized to develop integrated multi-organ platforms as an essential testing requirement during the advanced drug discovery steps. Nevertheless, there are major challenges hindering the development of predictive multi-organ models and their widespread applications for drug testing. Development of biomimetic multi-organ platforms based on principles of physiologically-based pharmacokinetic and pharmacodynamic (PBPK-PD) models is crucial for characterizing the metabolic activity of drugs upon their interaction with multiple organs.

In this review, we briefly describe the main principles of PBPK-PD modeling and discuss groups of tissues that can be adequate candidates for a particular PBPK-PD model structure. We further provide an overview of the design principles that can be extended to develop protocols for fabricating the next generation of predictive multi-organ platforms. Moreover, we highlight recent advancements in technologies of microfluidics and tissue engineering for the development of hanging drop organoids and organ-on-chip platforms for making multi-organ systems interconnected via engineered on-chip or off-chip vasculatures. We also discuss the challenges of developing biomimetic body-on-chip (BOC) models such as the scaling, cell types, selecting the suitable medium for all cell types, and optimizing the physiological parameters.

## 2. Drug Testing and Design of BOC

There is a critical need for the development of a functional approach providing organ–organ contacts to mimic the crosstalk of multiple organs upon testing drug candidates [16,17]. Critical considerations need to be implemented for generating a micro-physiological BOC model to replace existing organ systems and to attain a high degree of prediction for the disease modeling and drug discovery. A combination of cells simulating different tissues, cell-cell interactions, cell-matrix crosstalks, and a biomimetic exposure to disease conditions or toxins are some of these considerations [18,19,20]. For instance, it is important to provide appropriate cell-cell and cell-matrix interactions to simulate the regular beating of the heart and its perturbation upon exposure to toxins or diseases stimuli. The type of cells (single or multiple origins), the direct microenvironment of cells, the medium preserving differentiated states of different cells, as well as the three-dimensional (3D) configuration of tissues may all impact outcome measures. Further features to consider include dose–response characterizations and the developmental stages of selected cells [21,22].

To predict the response of whole-body to drugs, the concept of PBPK-PD modeling was developed [23,24,25,26]. The term “PK” refers to the prediction of time-dependent concentration of a substance in a living system. A PBPK model is made on physiological considerations, where the human body is separated into distinct compartments representing organs, connected by the universal blood circulation. There is a mathematical simulation method to investigate the interactions of multiple organs in a multi-organ system and to use a time-dependent drug concentration profile for studying the impact of drugs and metabolites on the body organs. A PBPK model gives a prediction of a drug concentration profiles and its metabolites from a given dose [27,28]. The term “PD” refers to the pharmacological (for example, the death of tumor cells in response to a chemotherapeutic agent) influence of a drug. The combination of pharmacokinetics and pharmacodynamics creates an integrated PK–PD model to predict the time-course pharmacological effects from a dosage. Several review papers are available which discuss integrated PK–PD modeling [23,24,25,26].

**Figure 1:**
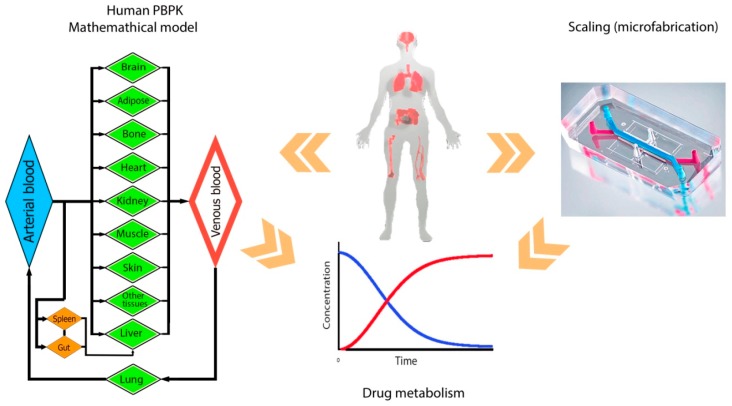
The design of multi-organ-on-chip platforms and the analysis of biological systems relying on the physiologically-based pharmacokinetic (PBPK) simulation. The drug action is recorded and used for the simulation process. The figure is modified from Reference [31].

The PBPK model is derived from the anatomical and physiological characteristics of organisms, and not from the drug-related data [29,30,31]. The major structural parts of this model are the physiologically realistic body tissues and fluids (Figure 1). The primary steps in developing a PBPK model is to determine the structure of the body model and its tissue components, writing the proper mass balance equations, and quantifying the parameters involved [32,33]. Mass balance equations describe the flow and metabolism for each organ, essential to building a PK model. To solve the ordinary differential equations (ODEs) for all compartments, physiological (the flow rate and the size of chambers), and enzyme-kinetic parameters should be determined [23]. These ODEs provide information about the uptake, distribution, metabolism, and excretion of a drug candidate such that a multi-organ platform can be realized as a physical representation of a PBPK model. To fabricate a multi-organ model based on the designed PBPK model, the capabilities and limitations of microfabrication need to be also considered.

The following combinations are several groups of tissues or organs that can be appropriate candidates for a particular PBPK model: (1) Core tissues/fluids/organs: the blood is subdivided between the venous and arterial vessels, the liver used as the central metabolizing organ, the kidney used for renally excreted drugs, and the adipose tissue used for lipophilic compounds; (2) Tissues with special interactions with a particular drug: this may consist of the eliminating tissues (intestines and lung), tissues with sites of drug administration (skin, gastrointestinal tract, and lungs), and tissues with possible sites of actions when the PBPK model is developed; (3) Tissues accounting for a substantial volume of distribution. Large body tissues with highly lipophilic contents such as muscle, adipose, skin, and bone are good candidates; (4) Tissues that are not included in the abovementioned classes but for which experimental data is obtainable. Using any other tissues that are not included in the groups defined above or any exclusion of those tissues entirely depends on their impact on the time-course of the mass balance for the whole body or for a particular drug [34,35,36].

Upon the preparation of PBPK model equations, parameters of the model need to be estimated. A data set made of concentration-time profiles for each organs/tissues/fluids is generated via experimental testing. The PBPK equations are then fitted numerically to the experimental tissue concentration-time data to provide predictive responses [37,38]. A particular software language can be used to code the PBPK equations. Typically, the parameters estimation of a successful PBPK model needs hundreds of runs and a repetitive and alternating incorporation of different numerical optimization methods [39,40,41].

BOC models should mimic physiologically-relevant interactions between organs. To achieve a predictive PBPK model relying on biomimetic data, some design principles have to be considered. This includes the ratio of cell mass (one cell to another), shear rates generated by the fluid flow, the ratio of free liquid volume to cell volume for each compartment, the time that each organ is exposed to a molecule (residence time), and the cell-related biological response in each compartment. Esch et al. [31] have set these criteria at the organ level. The primary criterion is the equality in concentrations of unbound critical nutrients and cytokines at the steady-state condition present in the human body. The equality is required between the time-dependent drug concentration profiles in the blood fluid and that in the human body. Also, time-dependent unbound drug concentrations for each organ in the chip should be equal to the one in the body. They used a mass balance for concentrations of compounds in the tissue and blood and then solved the equations. Consequently, a series of parameters and equations are proposed for the design purpose [27,30,42,43].

The application of existing PBPK models is usually limited by the difficulty of finding precise parameters of the model. Some required parameters include the organ’s volume, the ratio of cardiac output blood flow rates to each organ, the number of cells for each organ, as well as the residence times of each organ that can be found in the literature. However, the estimation of enzyme-kinetic parameters and clearance rates is mostly difficult. In some individual cases, in vitro enzyme-kinetic parameters are extrapolated to in vivo values. In vitro to in vivo extrapolation (IVIVE) method is usually used as an initial estimation of the in vivo clearance and release data if any newly developed drug is used [37,41]. Therefore, obtaining a set of experimental data is crucial to create a precise PBPK model. Unfortunately, researchers often deal with a limited access to numbers for essential parameters. PBPK modeling often relies on animal models, as there is a lack of human data [44]. Biomimetic physical multi-organ models are defined as the exceptional complement to the PBPK models reducing inconsistencies in the existing PBPK models due to their drawbacks in providing precise parameters.

## 3. Multi-Organs on Chip

Conventional 2D tissue models such as culturing in Petri dishes are still common in laboratory studies, but these cell assemblies are severely limited in mimicking cell-cell and cell-matrix interaction, and some other vital morphological and biochemical features [45]. Through the incorporation of a variety of technologies like microfabrication, tissue engineering, as well as cellular and molecular biology, novel biomimetic and high-throughput multi-organ models have been developed for the development of predictive models for drug discovery.

### 3.1. Organoid and Hanging Drop Spheroid Culture Models

Organoid (spheroid) cultures offer a body-like environment for testing drug toxicity. Organoids are 3D microtissues that can overcome major constraints of 2D tissue models and provide prolonged viability and function for cells. Organoid-based techniques are compatible with a variety of different pipetting methods and co-culturing techniques for the development of multi-organ models [46]. In these multicellular aggregates, the need to supporting gels or matrices is eliminated [10], the adverse influence of adhesion to artificial surfaces is removed [11], and therapeutic agents are more reliably experimented [12].

**Figure 2:**
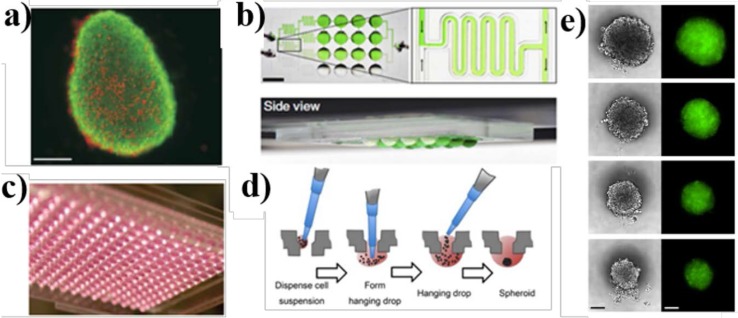
Organoid hanging drop cultures for 3D coculturing of single or multiple tissues. (**a**) HepG2 spheroid formed by the spinner flask method in day 10 of the cultivation, Bar = 100 mm [49]; (**b**) The top-view of the culture device and the side-view of the magnified laminar flow condition, Bar = 5 mm [10]; (**c**) 384-well plate for the formation of hanging drops [50]; (**d**) The spheroid’s self-assembly in wells after pipetting the cell suspension [50]; (**e**) Spheroids formed in the culture device illustrated in (**b**) 60 h after seeding, Bar = 100 μm [10].

Organoids are formed via several different methods, e.g., utilizing rotating cultures in roller tubes [47,48], spinner flask cultures (Figure 2a) [49], stationary cultures in hanging drops with well-known 96- or 384-well plates (Figure 2c,d) [50,51], and the growth on non-adherent surfaces [52,53]. These cell colonies are formed by self-assembly methods, thereby their production remains reasonably reproducible [54]. The hanging drop technique has been widely used for decades [10]. This technique can be implemented by pipetting drops of the cell suspension onto a substrate followed by culturing the suspension to form aggregates of a microtissue while settled at the bottom of resulted hanging drop (Figure 2d,e) [46]. This technique can enable a secure and controllable spheroid formation for the high-throughput screening of drugs [50]. In more advanced systems, organoids are formed and characterized either off-chip within conventional well plates and then manipulated by the pipetting [11,54] or cultured inside a microfluidic system, providing a flow of nutrients to cells [55].

Three-dimensional in vitro systems are capable of reproducing several key organ features observed in vivo such as the complex physical and chemical microenvironment, alteration in the cells composition and their interactions with the matrix, morphological features, and relevant cellular responses to highly model-dependent therapeutics [56,57,58,59,60]. Organoids have been used predominantly as 3D multicellular aggregates to study self-organizing stem cells [61], the embryonic differentiation [56], the liver [62,63,64,65], and the tumor [57,58,59,60]. Malignant tumor cells and other cells existing in the tumor microenvironment such as fibroblasts, endothelial cells, leukocytes, and hematopoietic cells can form organoid constructs [66,67,68,69]. Also human cancer organoids have been used to analyze the response to patient-specific antitumor drugs [54]. For instance, Markov et al. [70] developed a gravity-fed microfluidic-based organotypic culture platform for the long-term (about three weeks) culturing of human epithelial cells and characterizing their invasive and tumorigenic variants within the 3D matrigel matrix. In this thick-tissue bioreactor (TTB), the breast epithelial cell lines showed a noticeable morphogenesis pattern, forming hollow “mammospheres” in 3D gelled tissue matrix. The model was successfully used for the real-time staining and visualization of cells’ behavior and to studying the delivery of drugs like matrix proteinase inhibitors into the constructs, reducing the formation of mammosphere. This platform is promising for high-throughput testing of patient-specified drugs. In a very simplified but unique model for the development of a multi-organ system using organoid-based techniques, Frey et al. [10] conducted a functional analysis using a completely open microfluidic hanging drop system (Figure 2b,c). A combination of micro-tissue spheroids of human colon carcinoma cell lines (HCT-116 eGFP) and a rat liver micro tissue (rLiMT) resulted in a configurable hanging drop network. Such a model enabled the discovery of inter-organ signaling and the understanding of tissue-tissue interactions and extracellular matrix (ECM) specifications under highly controlled conditions. The remarkable idea of inverted substrate resulted in a high-throughput technique in studying the formation of multicellular spheroids. Towards a high-throughput spheroid-based multi-organ system, Kim et al. [51] established a flexible microfluidic platform compatible with standard 96-well plate formats in which up to 66 spheroids of various cell types were cultured using a parallelized culturing in a conventional incubator. The spheroids of rLiMTs and HCT116 eGFP were formed off-chip using the hanging drop method, directly placed into the microdevice using gravitational forces, and subsequently connected to each other through the medium perfusion in micro-channels. Although the culturing method is simplistic, it remains difficult to study the interconnection of multiple micro-tissue types over a hundred parallel experimental conditions.

Although promising for diverse applications, organoid-based hanging drop platforms have some fundamental limitations that hamper their further sophisticated progression. Organoids may not have equal diameters and this increases uncertainty [10]. They are disorganized aggregates of multiple cell types constituting spherical shapes and cannot exhibit many functional properties of tissues. Thus they cannot be extended to fabricate organ models. Positioning and patterning of cells or ECM proteins are not defined in organoid models which are crucial to the proper function of multiorgan-level culture models. The small volume of organoids and the complexity of sampling from the luminal content hinder investigations on several functional activities of organs in the absence of circulating fluid flow such as the absorption, secretion, and transcellular transport [71]. Cells are entrapped and thereby it is not straightforward to carry out functional analysis of cells through harvesting cellular components [10]. Lacks of fluid flow, fluidic shear stress, and in vivo-like mechanical cues also obstructs the applicability of these models. Microfluidic-based spheroids can considerably overcome these shortcomings of conventional organoids-based hanging drop methods via providing continuous perfusions and configurable gradients in a controllable manner. They also provide numerous advantages such as improvement in the cell environment, spheroids formation, viability, etc. [45,72].

### 3.2. Microfluidics: A Proficient Framework for Multi-Organ Studies

Microfluidic devices provide tissue-tissue interfaces as a key factor in producing reliable in vitro organ models, thus they can be used to replicate human pathological conditions [73]. The existence of media perfusion is one major advantage of microfluidic systems [74], which allows supplying essential nutrients and oxygen to tissues in physiologically-relevant residence spans. The sample volume derived from the microfluidic device is considerably reduced, overcoming the constraints of patient-derived samples. The ability to circulate the medium in a closed-loop system and the optical transparency of microfluidic layers also allow the integration of various biosensors for the real-time monitoring of cells or tissue-specific responses. Moreover, microfluidic devices have also the ability to incorporate different organs in the same setup called “multi-organ model on a single chip”. Organ-on-chips have specific features that make this technology a climax in the field of multi-organ physiology, disease modeling, and drug discovery. Such microfluidic platforms can thus yield unprecedented levels of organ functionality as a result of distinct advantages over conventional 2D or 3D platforms.

**Figure 3:**
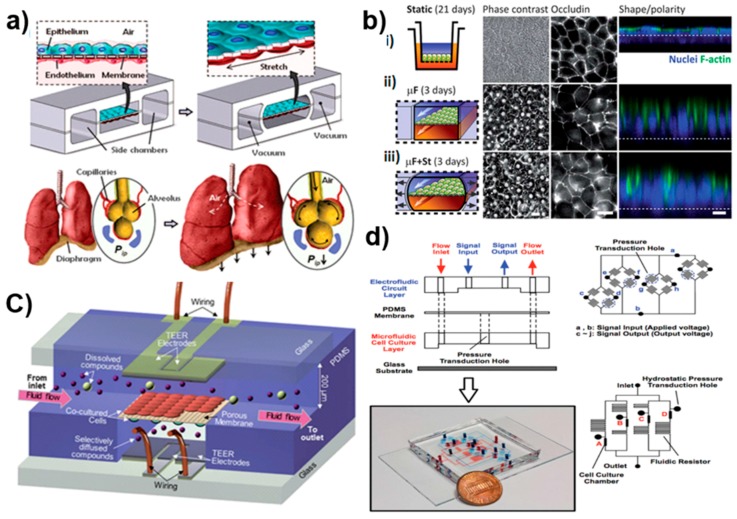
Examples of sophisticated organ-on-chip applications. (**a**) Compartmentalized polydimethylsiloxane (PDMS) microchannels for breathing activities in the lung. A thin, porous, and flexible PDMS membrane coated with the extracellular matrix (ECM) forms an alveolar-capillary barrier. The device recreates physiological breathing movements by applying a vacuum to side chambers and leads to mechanical stretching of the PDMS membrane to form the alveolar-capillary barrier. The inhalation in the living lung contracts the diaphragm and reduces the intrapleural pressure and physical stretching of the alveolar-capillary interface [78]; (**b**) The morphology of epithelial cells cultured in the (i) static Transwell system for 21 days, gut-on-a-chip with a microfluidic flow without (ii) or with (iii) the application of cyclic mechanical deformation for three days. The schematic layout of (**left**); fluorescence views (**center**) of the occludin as the tight junction (TJ) protein, and the confocal fluorescence views (**right**) of the epithelium (nuclei in blue and F-actin in green) [80]; (**c**) The design of the developed microfluidic blood-brain barrier (BBB) with integrated electrodes for measuring the trans-epithelial resistance across the barrier [98]; (**d**) The microfluidic cell culture device with embedded electrofluidic pressure sensors. The PDMS membrane is sandwiched between two other PDMS layers: an electrofluidic circuit layer and a microfluidic cell culture layer. The layout of the electrofluidic circuit layer for pressure sensing at four locations, and an equivalent Wheatstone bridge circuit of the pressure sensor [84].

Biomimetic physical forces are provided in microfluidic chips using fluid forces [75,76,77], cyclic mechanical strains [78,79,80,81], and mechanical compression [82,83], crucial for replicating the in vivo-like biophysical microenvironment. The accurate control of fluid flow and shear stress over cells improves their long-term viability [76,78,84], essential for conducting experiments on tissues in a clinically-relevant time scale [85]. In addition, microfluidic chips can accommodate physical and chemical gradients in a diverse set of studies such as chemotaxis [86,87], axon outgrowth [88], differentiation [89], and cardiac tissue formation [90]. The corresponding shear stress can be adjusted by changing flow rates or channel dimensions [75,76], by using membranes to separate cells from the flow [75], or by constraining the cell passage with micro-engineered substrates [91]. The microchannel geometry highly impacts the nutrient and oxygen delivery to tissues, and thus influences cell survival, proliferation, and differentiation [75]. A low amount of fluid shear stress improves the delivery of drugs to cultured cells and produces biomimetic toxicity responses in primary human epithelial cells [92]. The presence of fluid flow also provides feasibility to characterize interactions of tissues with circulating cells or molecules such as blood cells, immune or tumor cells, drugs, or other chemicals [93,94]. Flexible side chambers combined with cyclic suction provide cyclic mechanical deformation by periodically stretching and relaxing the walls and the central membrane. The simultaneous exposure to rhythmic mechanical strain and fluid shear stress, similar to in vivo conditions, enhances organ-specific functions [71,82]. This has the potential to mimic activities such as breathing (Figure 3a) [78,79], cardiovascular contraction and pumping, as well as peristalsis cycling in the gut (Figure 3b) [80].

Contrary to organoid models, microfluidic-based organ-on-chips can employ various patterning methods like ECM micropatterning [95,96], micromolding techniques [97], and laminar streams positioning [86], to precisely position one type of cells within the microchannels consistently next to other type of cells [78]. The organ-on-chip platforms are also well-suited for investigating tissue-tissue interactions through culturing multiple cell types positioned separately in several microchannels while interconnected by membranes or porous substrates [78,79,98]. The transcellular mechanisms of transport, the degree of protein secretions, and the ion absorptions for various cell types in addition to the tissue barrier performance such as the blood-brain barrier (BBB) or epithelial barriers are explored in such devices [78,80,98,99,100,101] (Figure 3c). Multiple cell types from different organs crosstalk via fluidic channels that promote the organ-organ interaction and enable examination of the drug transport and distribution.

Improving the functionality of conventional macro-scale 3D culture systems adds to the complexity of the system, thereby characterizing the function of living organs is inconvenient and needs advanced imaging facilities. Instead microfluidic devices are made of transparent materials like polydimethylsiloxane (PDMS) that enable a high-resolution and on-line dynamic monitoring of the metabolic activity of cells. This is implemented via integrating optical or electrical detection systems like confocal fluorescence microscopy, microfluorimetry, amperometric, and multiple electrode arrays (MEA) into microfluidic devices [102,103,104,105,106,107,108,109,110]. Microfluidic systems are thoroughly micro-engineered models, thus they can be incorporated with miniaturized biosensors like optical, electrical, and electrochemical sensors [111,112,113], to conduct real-time detection of chemical substances of interest expressed by cells, together with physical, electrical, and metabolic activities of cells [114], to investigate cell migration [115,116,117], drug delivery [118], hydrostatic pressure, and shear stress (Figure 3d) [84].

Stem cells differentiation is highly appealing owing to the promise for modeling diseased organs. Microfluidic platforms provide comprehensive supervisions on many parameters of in vitro culture system such as the physical and mechanical forces, direction, and position of cells of diverse tissues, chemical gradients of drugs or nutrients delivered to cells, tissue-tissue interactions, and the process of differentiation. Biophysical properties such as geometric cues [119] provided within microfluidic chips have proven advantages in lineage differentiation [120] and gene expression profiling [121,122]. This significant advantage is also combined with the high-resolution in-line monitoring which altogether demonstrate the feasibility of replicating individual functional components of organs. A considerable number of studies used microfluidics to regulate the differentiation of embryonic [121,123,124], mesenchymal [119,125], and neural stem cells [126,127,128]. Given the recent advances in stem cell differentiation on-chip, this field of study proposes a prospective view of a human-on-a-chip with a large number of organs originated in a single patient [71]. With incorporating novel vasculature systems developed specifically for organ-on-chip platforms [94,129,130,131], it is currently possible to interconnect organ units to construct a functional multi-organ system and ultimately a whole body.

Last but not least, the assessment of drug toxicity and efficacy has remained a persistent issue to address, given the fact that animal models have failed to provide consistent results with human drug testing, where the absorption of drugs differ due to species dissimilarities in the membrane transport [71]. Furthermore, static in vitro cell cultures cannot maintain metabolic activities for the relevant lifespan [132]. The integration of liver tissue as one essential organ for toxicity examination resulted in development of appropriate models for investigating drug transport [133,134,135] and toxicity assessment [132,136]. Also tumor-specific cellular responses to particular drugs were screened in several cancer-on-a-chip systems [137,138]. For instance, the exposure of chronic myeloid leukemia single cells to the tyrosine kinase inhibitor dasatinib (Sprycel) demonstrated a satisfactory treatment [137]. Lung cancer cell lines subjected to diverse concentration gradients of chemotherapeutic agents also contributed to the individualized drug sensitivity screening [138]. In addition, microfluidic organ-on-chips are suitable for assessing drug efficacy in a high-throughput platform, where multiple tissues are examined simultaneously on a single chip [95,139].

Despite all advantages of microfluidic devices in drug toxicity and efficacy testing, there are factors that limit the usage of these devices by both non-engineer researchers and pharmaceutical companies. Microfluidic platforms are presently sophisticated and multiplexed as they work with syringe pumps and pneumatic fluidic handling systems. This requires great skills in fluid and cell manipulation in the microscale, which is incompatible with biologists or clinicians needs of user-friendly well-plate and pipetting toolkit-based approaches. The demand for a reliable microfluidic technology applicable for biologists and clinicians contributed to the idea of “modular microfluidics”. In modular microfluidic systems, the non-expert end-user can build the desired custom device using microfluidic assembly blocks (MABs) in a convenient and reliable manner without a need for a complex designing software or a cleanroom facility [140]. Well-plate microfluidic devices—integrated multiple bioreactors in a multiwell format—are modular devices providing a straightforward interaction of multiple organs on chip. Several modular microfluidic models have been reported in both academia including the microfluidic breadboard for integrated biochemical analysis [141] and assembly blocks [142], as well as industry by some commercial vendors [140]. The modular architecture is portable and thus can be adapted to standard laboratory procedures for frequent transfer between workplaces such as the cell culture bench, microscope, and incubator [140]. The principal motivation for further improvements and developments in the field of modular architecture is to enable the integration of these modular devices into established pharmaceutical industry workflows. This contributes to the drug development process by filling the gap between prospective users and technology developers in this field of research [143].

### 3.3. Microfluidic-Based BOC Models for Drug Development

Presenting a new drug to the marketplace currently consists of prolonged and expensive procedures of drug discovery, drug development, clinical researches, and goal-oriented marketing [144]. In the drug discovery stage, depending upon the related disease, the first step is to identify the specific drug target, for example, a receptor or ion channel in cells, a nuclear receptor, deoxyribonucleic acid (DNA), ribonucleic acid (RNA), or even an unknown target. The second step in drug discovery is to confirm whether the drug has the intended impact on the disease. This step is carried out through sophisticated experiments in vitro on living cell cultures or via animal models. Upon the validation and identification of the target, it is exposed to a large number of compounds in a high throughput screening (HTS) mode to find the lead compound that acts on the confirmed target. In the drug development stage, the drug toxicity testing and pre-clinical studies involving pharmacodynamics and pharmacokinetic are conducted in vitro or in vivo on lead compounds for crucial assurance about the safety and performance, necessary in further clinical trials. The last stage is the clinical trials where the compounds that are successfully proven safe and effective are tested on human subjects to assess their performance for the corresponding disease. After accomplishing all these three stages properly, pharmaceutical companies may file the new drug application [145].

The extensive process of pre-clinical testing and validation is ineffective, expensive, and time-consuming so that merely one or two out of ten drugs that enter clinical trials are approved for further human use [146]. Current experimental drug screening methods cannot produce reliable pre-clinical outcomes which severely impedes drug development progress [147]. Costly and highly repeated late-phase drug attritions are the principal reason for unprecedented challenges hampering pharmaceutical progression including scientific, economic, and legal issues, dubbed the “pharmaceutical industry grand challenge” [148]. Pharmacological profiling assays proposed by four major pharmaceutical companies are examples of advancements in developing predictive models with a reasonable reproduction of human metabolism [149]. This may elevate the success rate of clinical trials and reduce the cost of drug development. The primary focus in pre-clinical models is on using absorption, distribution, metabolism and excretion (ADME) methods and other prediction and validation tools as an upgraded technology for the rapid recognition of adverse side effects or failure of pharmaceutical candidates [149]. BOC platforms are promising to develop reliable in vitro platforms with the desired anticipation ability of human- or individual-specified drug-performance. In the following sections, we discuss in detail the design, particular features, and the performance of several multi-organs on-chip platforms developed for testing therapeutic candidates [31,150].

### 3.4. Two Organ Models

Single organ-on-chip platforms have demonstrated the significance of cell-cell and cell-ECM interactions in 3D environments. However, these systems cannot simulate the interplay between different organs that are physically separated but their interactions through the circulation system are necessary for their proper function. As discussed in Section 2, several organs in the human body such as the liver, heart, intestine, kidney, and skin are primary targets of the drug toxicity and allergic reaction studies. Therefore, several microscale co-culture platforms for investigating the metabolism and toxicity of drugs have been developed during the past few years, while very few of them have followed the principles of PDPK modeling properly [23,27].

#### 3.4.1. Liver-Heart Co-Culture

Of different multi organs platforms, liver-heart interactive micro tissue systems are quite important as they hold a great promise in investigating the toxicity of many cardiovascular drugs that are eventually metabolized in the liver. Such platforms can be designed to understand the micro physiological interactions between the heart and the liver in the micro scale to further model the response of these organs to numerous drugs and to study their efficacy. For instance, Vunjak-Novakovic and her co-workers successfully developed an integrated liver-heart micro scale platform from human pluripotent stem cells for drug testing [151]. This platform was able to direct the differentiation of human PSCs into cardio myocytes, endothelial cells, and hepatocytes in a controlled fashion. It was also shown that the differentiated cells were able to become mature cells by using physical cues for cardiomyocyte formation and chemical cues for hepatocyte formation. These cells were eventually incorporated with each other to form cardio and liver micro tissues. Following the importance of developing microtissues of liver and heart, and also the use of PSCs in developing a platform to develop the liver and heart cells on-chip, many groups have taken the advantage of using induced PSCs (iPSCs) to create functional constructs [152]. Mathur et al. [153] reviewed the advantages of using iPSCs to develop myocyte-hepatocyte interconnected systems that could potentially reduce the cost of animal models for drug testing assays.

#### 3.4.2. Liver-Skin Co-Culture

Skin and liver are important targets of drug toxicity studies. The human skin is one primary target tissue for cosmetics and drugs that are administered transdermally [154,155]. The liver has been extensively considered as the prime organ for drug toxicity analyses as most of the drug metabolisms occur in the liver [71,156,157]. The co-culture of skin and liver enables the investigation of the toxicity and efficacy of the systemic administration of drugs.

**Figure 4:**
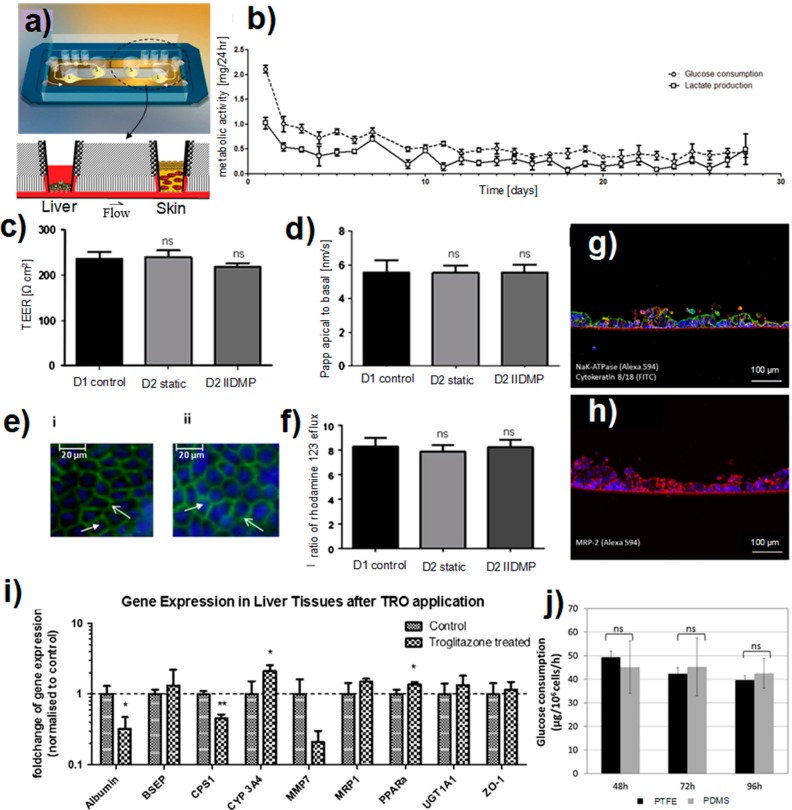
Multi-organ on-chip platforms for the disease modeling and drug studies. (**a**) The 3D schematic of the liver-skin co-culture microfluidic device; and (**b**) The metabolic activity of the co-culture of liver and skin [158]. Analyzing the integrity and functionality of the intestinal barrier after 24 h of dynamic co-culture; (**c**) Transepithelial resistance (TEER) measures; (**d**) The apparent permeability (Papp) to the Lucifer yellow (**e**) and the staining of tight junction components: (i) occludin and (ii) claudin. The tight junction (open arrows) components were stained in green by specific antibodies and the nuclei (closed arrows) in blue by 4′,6-diamidino-2-phenylindole (DAPI); (**f**) The Pgp activity was investigated by the measures of efflux ratio of rhodamine 123. The results obtained after one day of the static culture in Petri dish (D2 static) or the dynamic co-culture (D2 IIDMP) are expressed relative to the control consisted of the static culture of Caco-2 TC7 after 21 days (D1 control) [13]; (**g**) Staining of the small intestinal epithelial tissue for the transporter NaK-ATPase (red) and cytokeratin 8/18 (green); (**h**) Staining of the small intestinal epithelial tissue for the ATP-dependent export pump MRP-2 (red); (**i**) qRT-PCR data of liver tissues from the control (normalized to 1) and treated co-cultures analyzed for the expression of albumin, BSEP, CPS1, Cyp3A4, MMP7, MRP-1, PPARa, UGT1A1 and ZO-1 [14]; (**j**) The glucose consumption of Madin Darby Canine kidney cells cultured in dynamic biochips made from PDMS and PTFE [15].

A liver-skin on-chip model was developed within a microfluidic device for the long-term cultivation of cells [158]. The microfluidic device was composed of the skin (S) and liver (L) compartments that were interconnected through a microchannel (Figure 4a). The air-liquid interface for the skin tissue was achieved by standard Transwell^®^ inserts. Microtissue aggregates were used as liver tissues while human biopsy tissues were used in the skin compartment. The platform enabled the co-culturing of liver and skin tissues for 28 days without compromising their function and viability (Figure 4b). Moreover, tissues crosstalk was demonstrated as the skin consumed the liver-produced albumin. The platform was used to study the toxicity of troglitazone as an antidiabetic and anti-inflammatory drug prescribed for patients with diabetes type 2. The results exhibited a dose-dependent response of this in vitro model to the drug candidate at the RNAs level. To mimic the human vasculature, the microchannels connecting the two tissues were coated with human dermal endothelial cells [14]. The endothelial lining interacted with other tissues via transporting tissue-tissue signals and recruiting leukocytes into a region of local damage in the organism.

Liver-skin co-culture platforms are suitable for investigating the drug absorption and metabolism in systemic delivery modes. Maschmeyer et al. [14] studied the exposure of a liver-skin model interconnected through an endothelialized microchannel to the troglitazone. They performed daily treatments of the two-organ model for nine days by adding a solution of troglitazone (50 µM) to the skin barrier. The results supported the capability of such a microfluidic platform for drug studies.

#### 3.4.3. Liver-Intestine Co-Culture

The absorption of orally administered drugs through the gastrointestinal system has been the subject of many studies during the last decades [159,160,161,162]. Along with the liver, the intestine plays a major role in regulating the extent of absorption of orally administered drugs [163]. Moreover, since the human intestine accommodates a diverse community of microbes that contribute to the metabolism and digestion any change in the homeostasis of the intestine can lead to disorders including liver diseases. Therefore, it is crucial to develop in vitro models that can mimic the interaction between the intestine and liver for drug studies and disease modeling.

Recently, a microfluidic model that imitated the organ-organ interaction between the intestine and the liver was developed using polycarbonate cell culture inserts [13]. Intestinal Caco-2 TC7 and HepG2 C3A cell lines were used in the intestine and the liver compartments, respectively. The fluidic system comprised three similar co-culture units that were perfused simultaneously. Prior to each experiment, Caco-2 TC7 cells were cultured on polycarbonate inserts for 21 days to form the intestinal barrier. The culture experiments were then performed for three days under static and dynamic culture conditions. Comparing the static and dynamic culture of the intestine compartment showed that junctions were well established in both culture conditions. Moreover, the detection of Pgp efflux, and the transport of Lucifer yellow in the dynamic culture revealed that the functionality of the epithelial layer was similar to the static culture (Figure 4c–f). The interaction of intestine and liver compartments in the microfluidic device resulted in a significant upregulation of CYP1A enzyme activity in the liver.

The liver-intestine microfluidic systems are promising platforms for drug studies. In two recent studies, the dual-organ model was used to mimic the oral administration of drugs in humans [164]. Brick and coworker [13,14] investigated the first-pass metabolism of omeprazole and phenacetin in an intestine-liver model. Phenacetin reduces pain and fever and is metabolized to paracetamol by CYP1A2 of cells [165]. Omeprazole is used in the management of gastroesophageal reflux disease and is metabolized by CYP3A4 and CYP2C19 [166]. Their results showed a rapid absorption of phenacetin and omeprazole by the intestinal barrier in the co-culture device. In another study, Maschmeyer et al. [14] simulated the oral administration of troglitazone into an intestine-liver model. The results showed that the repeated dose testing did not affect the polarization of the intestine barrier (Figure 4g,h). Moreover, the liver compartment responded to troglitazone properly, as evidenced by the gene expression data (Figure 4i).

#### 3.4.4. Liver-Kidney Co-Culture

The kidney plays an important role in maintaining our general health and well-being. The kidney is responsible for filtering about 200 L of our blood each day. It is also responsible for producing and regulating several hormones and enzymes which help to control the blood pressure, create red blood cells, and maintain strong and healthy bones. The kidney is a primary target for drug-induced toxicity as it is responsible for the excretion of toxins that are not metabolized in other organs such as the liver.

Microfluidic organ-on-chip platforms enable an exquisite control over temporal and spatial cell growth and organ-specific metabolic functions. With these platforms, the systemic interaction of different organs such as the liver and kidney can be investigated. Shintu et al. [167] developed a liver-kidney co-culture system to investigate the toxicity of ammonia (NH_3_) as an environmental pollutant leading to metabolic acidosis and toxicity in the liver and kidney; dimethylsulfoxide (DMSO) as a free radical-scavenging solvent; and Acetaminophen (*N*-acetyl-para-aminophenol; APAP), or paracetamol as a hepatotoxic analgesic drug. The microfluidic device was able to capture essential metabolic end points for the toxicity screening of a wide range of small molecules. In another study, Jellali et al. [15] fabricated a liver-kidney co-culture microfluidic device using perfluoropolyethers (PFPEs) and PDMS materials. Polytetrafluoroethylene (PTFE) was proposed as an alternative material for overcoming some challenges associated with PDMS chips including the non-specific adsorption of proteins. Microchips made in PTFE and PDMS showed a similar response in terms of the cellular interaction of the kidney and liver compartments (Figure 4j). However, PTFE offered several advantages including a simple fabrication process and a minimal absorption of small biomolecules.

### 3.5. Multi-ORGAN Models

Multi-organ chips offer a number of advantages over traditional in vitro culture systems [31]. Virtues include the reproducibility of co-culture, the ability to alter its properties by controlling the fluid flow, and tailoring the communication among multiple organs which are more likely to be close to the actual human physiological conditions. In particular, the combination of different tissues played a key role in representing the function of several organs and understanding their communication as well as monitoring their behavior once exposed to a specific drug [31,168]. Also one way to regulate the fate of cells is to control their interactions with other cells and tissues in the microscale, shedding light on understanding how a specific drug can regulate the communications between multiple organs.

New pre-clinical trials evaluate the application of multi-organ chips as a potential way to model 3D tissues derived from many different cell sources and to understand the etiology of disorders [19,168,169,170]. Such devices hold a great promise in developing novel therapeutics, predicting the safety and efficacy of drugs, and understanding cell-cell and tissue-tissue interactions for developing novel clinical studies [19,168].

Examples include the co-culturing of the human liver microtissue, epithelial cells, neurospheres, and intestinal tissues. Wagner et al. [168] successfully co-cultured human liver microtissues with skin biopsies on a multi-organ-chip for two culture times, 14 days and 28 days. In another study, Materne et al. [169] successfully demonstrated the success of co-cultured liver micro-constructs and human neurospheres exposed to the media flow for two weeks. Maschmeyer and co-workers [19] developed a multi-organ-chip platform culturing different human 3D organ equivalents such as liver spheroids with skin punch biopsies, and neuronal spheroids or intestinal tissues at homeostasis over 28 days for repeated dose substance testing. Renal proximal tubule cells were seeded within a membrane to model the kidney as a combined approach with a skin biopsy. Over the entire 28 days of culture, all tissues showed a significant level of cell viability [19].

Interconnecting the organs on the multi-organs on-chip can be very challenging due to the complexity of inter-organs and intra-organs communications. Blood vessels on-a-chip can bring a deep insight into the understating of such communications. Zhang et al. [171] used PDMS tubes functionalized with human umbilical vein endothelial cells (HUVECs) to construct elastomeric blood vessels. Overall, this presented a body of works that demonstrate the significance of multi-organs chip platforms for studying the fate of cells and microtissues’ interaction into the appropriate phenotypes and functions, necessary to regain possible understanding of actual human physiology.

## 4. Challenges and Future of Multi-Organ Systems

Advancements in tissue engineering and microfabrication have led to the construction of organ-on-chip devices attempting to imitate fundamental features of a specific organ. Researchers combined the mechanical [172,173], biomaterial [174,175], and topographical [176] characteristics on in vitro models to recreate in vivo functionality as a novel approach towards drug testing. Since researchers are ever pursuing notable success in this area, it is not beyond the realm of possibility to reach the ultimate goal of fabricating a patient-specific BOC platform that can reduce costs of clinical therapies and eliminate their side effects [177]. The next mission, after remarkable studies on artificial heart [178], lung [78] , kidney [92], blood-brain barrier [80], gut [80], muscle [139], and liver [80] on chip, is to link these single-organ devices to make BOC platforms and utilize stem cells for the aim of developing patient-specific systems. The other approach to building BOCs is to incorporate organ compartments on a single chip.

Scientists, however, have to overcome numerous and arduous challenges to fabricate BOCs. Besides major challenges regarding the scaling and cell types discussed in this section, selecting the suitable medium for all cell types, simplifying the physiological parameters and industrial aspects demand careful attention. Delving further into the issue, each cell type requires specific growth factors and medium. Therefore, BOC’s medium should contain all the required nutrients of the organs. To elucidate, various approaches may be used such as combining different mediums that decrease the individual concentration of nutrients or using human blood which increases the possibility of undesired immune reactions [80]. The interconnection of multiple organs brings complexities that are not possible to tackle without considering some degree of simplification, thereby only essential physiological parameters should be incorporated [78,179].

For an organ-on-chip platform, biophysical and biochemical properties of the scaffold and the cell arrangement should be similar to natural ECM. The scaffold should supply an appropriate type of perfusion to cells. The literature has shown metabolic and functional differences between 2D and 3D tissue models [180,181]. while ordinary 2D studies maintain the original function and phenotype of cells, they still need to generate a truly “organ-like” morphology to be able to produce a biomimetic functional performance [182]. Recent advances in 3D patterning of cells in the microscale is promising to produce the next generation of “organ-like” organ-on-chip platforms to achieve an environment where cells act in vitro as they do in vivo [183,184].

### 4.1. Engineering Challenges

While multi-organ chips have many advantages to be used for pharmacology, physiology, or systems biology, there are a number of challenges that should be addressed to simulate the organ–organ regulation and drug–organ–organ interactions. Some engineering challenges in designing human-on-a-chip systems are to determine the volume of perfusion medium and the proper size of each organ, developing vascularized organs, controlling coupled organ systems, developing a common blood surrogate, and reducing the organ cost [185].

A human-on-chip needs to be designed to optimize the volume-to-cell ratio, and control how cells alter the surrounding medium to reduce the dilution of metabolites, as well as paracrine and autocrine factors. Engineering the issues associated with the dilution of sample volume and the analysis of small microfabricated bioreactors are primary implications. One advantage of microfluidic multi-organ systems is to support biomimetic volume-to-cell ratios considerably closer to physiological values. This can avoid the dilution of paracrine and autocrine factors, and other signaling molecules and metabolites. Also, the dilution of drugs or toxic metabolites into an excessive volume of medium might affect dose–response studies where the active compound is not the drug or toxin but a product of cell metabolism or signaling. Small volumes of microfluidic channels face issues with bubbles that are essential to tackle. The effects of evaporation, density, surface tension, and viscosity of the medium have to be considered. In small volumes, the surface binding of metabolites or drugs to a microfluidic chip or an analytical instrument can considerably affect concentrations. Maintaining the proper tissue to fluid volume ratio is an important parameter for designing a human-on-chip platform. In such a system, it is challenging to produce compact organs with both appropriate chronological responses and the capability to react to circulating cytokines. The adjustment of chemical concentrations in the perfusion medium is one of the major implications of small fluid volumes in a milli- or micro-human systems. An example of this issue is the injection of small volumes of precisely mixed fluids for simulating humoral control. It is non-trivial to connect the fluidic droplet injector into the circulatory system of human-on-chip since it can handle ultra small volumes (nanoliters to picoliters) [20].

Among different materials used for biomedical applications, synthetic polymers play an important role in developing multi-organ chips. Synthetic polymers offer a number of advantages over natural polymers. Of the most commonly used synthetic polymers in developing microfluidic chips, PDMS has shown an excellent degree of biocompatibility as well as appropriate chemical, optical, and mechanical properties such as permeability, transparency, and softness. A huge body of work exists to highlight the clinical importance of PDMS [81,83,102,103,104,186]. Although PDMS has promising properties, there are some disadvantages such as the non-specific adsorption of molecules, the release of uncrosslinked toxic PDMS molecules, and the absorption of less hydrophobic molecules [187,188,189]. Alternatively, people have used other polymers that are commonly used in designing microfluidic chips with their own advantages and disadvantages. Examples include PTFE, polymethyl methacrylate (PMMA), perfluoropolyethers (PFPEs), polyimides, SEBS, and thermoset polyesters [15,122,190,191,192].

### 4.2. Scaling

One primary concern of labs-on-chip, especially BOCs, is to reliably replicate the in vivo environment to achieve physiological responses from in vitro tests. The scaling as an important BOC designing characteristics plays an important role. Obviously, a disproportion scaling among various organs in a BOC model yields false results, particularly where their functions rely on organ-organ interactions [177]. To shed more lights on the topic, the liver is responsible for converting Tegafur, as a chemotherapeutic agent, to 5-fluorouracile [193]. As a pre-clinical test, the liver-on-chip platform needs to be sufficiently scaled to metabolize a considerable amount of Tegafur to 5-fluorouracile for the tumor treatment. Furthermore, in the case of simulating a large tumor or a small liver, the experiment may lead to false results [177]. Allometric scaling and residence-time based scaling are two primary approaches to incorporate the impact of scaling in a multi-organ model.

The *allometric* science that correlates the body mass (*M*) with physiological parameters (*Y*) is defined as [194,195]:

*Y* = *a* × *M^b^* (1)

where “*a*” is a constant depending on a parameter and “*b*” is a scaling exponent. The *b* value differs in sign and quantity depending on the relation of parameter and mass. When *b* = 0 the parameter (such as the bone density in mammals) does not alter by mass and *b* = 1 (cell number) shows a direct relation between the parameter and body mass. Until *b* has a value between 0 and 1 (*b* = 0.75 for the metabolic rate and *b* = 0.25 for lifespan), the rate of increment for the parameter is less than the body mass. Whenever *b* is greater than 1 (*b* = 1.33 for bone mass) the relation is vice versa. A negative value represents a reduction in the parameter (*b* = −0.25 for the respiratory frequency). This approach is utilized to assess whether the downscaled environment keeps the original relationship among diverse tissues [196].

Allometric scaling may not be authentic for all type of organs [197,198]. The allometry equation is based on three critical assumptions that should be considered in designing BOC platforms. That *b* = 0.75 for many physiological parameters such as the basal metabolic rate (BMR) is with regard to the assumption of “space-filled” and “natural selected” transport networks. While most of the organs-on-chip are not space-filled and not optimized for supply distribution, the three-quarter scaling does not fit for BOCs [195]. Furthermore, designing organs-on-chips is based on allometric calculations that presume individual cells produce the same amount of energy that they produce in vivo [199]. The allometric calculations also assume fixed volume and BMR, while in vivo BMR is usually higher than in vitro [200]. Finally, nutrients and oxygen availability not only differ in animals (heart rates for mice and human are 600 and 80, respectively [177]) but also vary with in vitro experiments. The impact of oxygen accessibility in cell responses may be a reason that higher BMR observed in smaller species [201]. Overall, researchers should understand the principle and philosophy of allometric scaling before applying it.

Researchers use allometric scaling to investigate desired physiological parameters through their specific multi organ-on-chip. Ucciferri et al. [196] designed a multi-compartment modular bioreactor (MCmB) to scale hepatocytes with endothelial cells. They compared the cell number scaling model (CNSM) and the metabolic and surface scaling model (MSSM) to consider the ratio of the hepatocytes chamber with the endothelial cells chamber. The results presented an optimal 4:1 ratio for hepatic-endothelial chambers. Since the cell number plays an important role in regulating the filtration and absorption rate of drugs, CNSM approach revealed 1:36 ratio for the chambers. This study demonstrated the adversity of selecting appropriate physiological parameters for a BOC system. Details of scaling rules and challenges have been extensively discussed by others [202,203,204,205].

### 4.3. Cell Sources: Cancer Cells versus Stem Cells

The selection of an appropriate cell type is key to any tissue engineering research. The three main types of cells are the primary cells, immortalized cells, and stem cells. Primary cells are directly extracted from the tissue with no need for adjustment, while their preserving, culturing, and extraction for in vitro experimentation are challenging. Immortalized cells instead can divide for a long time as a result of a natural or intentionally-induced mutation. While these mutated immortalized cells may change their functions and phenotypes compared to primary cells, they are widely commercialized and accessible. Stem cells with the potential for differentiation to various cells can recreate in vivo environments which make them a prime candidate for organ-on-chip technology [206].

Incorporating stem cells into organ-on-chip platforms propounds a bright future for tissue engineering and aligns with the ultimate goal of current organ-on-chip studies in creating patient-specific BOC platforms. Human induced pluripotent stem cells (hiPSC) have revealed functional resemblance with different cell types in the in vivo environment [207]. For instance, hiPSCs and animal cells were compared in function for various organs such as heart [208], liver [209], lung [78], kidney [210] and brain [211]. In a notable study, Takayama et al. [209] utilized hepatocyte nuclear factor 4α (HNF4α) to establish the protocol for differentiating hiPSCs to hepatocytes. The uptake and excretion of indocyanine green (ICG), the uptake of low-density lipoprotein (LDL), and the storage of glycogen were essential characteristics of hepatocytes that transduced stem cells revealed [209]. Recently, hiPSCs derivatives within microfluidics became popular among research groups to spatiotemporally differentiate these cells inside the chip, and optimize the delivery of exogenous factors and remove cell-secreted factors, for applications like vascular modeling and drug screening [101,117,212,213]. It seems hiPSCs could play a vital role to achieve the ultimate goal of organ-on-chip systems. However, some enhancements in scaffolding, culturing, differentiation, and sensing are still required.

### 4.4. Computational Bioinformatics Opportunities for Drug Design in Multi-Organ Platforms

Recent studies have shown that small molecule drugs can bind to proteins involved in entirely different pharmacology [214,215,216]. Interestingly, 35% of known drugs are reported to act against more than one target [217]. Such observation can be both challenging and promising. Challenging, because such molecules can lead to adverse drug reactions resulting in failure of the drug and thus need toxicity studies for drug candidates [217]. Promising by taking into account the fact that we can inhibit multiple targets by a single drug to increase the treatment efficiency [218] or to treat more than one disease [5,6]. Such evidence has led to a well know strategy that one can use an already existing drug for another disease to cure more diseases with a single drug, also called drug repositioning [6]. Different approaches like protein-ligand interaction network analysis [214,215,216,219,220] or chemogenomics analysis [221,222,223,224,225,226] have been utilized to study drug repositioning opportunities in the process of drug discovery. Computational techniques that have been developed to facilitate the drug repositioning procedure could be categorized as either drug-based or disease-based methods [227]. In the drug-based approaches, the chemical perspective of drug molecules is considered such as the structural features [227,228,229,230], the biological action of drugs [231,232,233,234], and the molecular docking [235]. On the other hand, in the disease-based methods, the clinical perspective or pathology of the disease is considered as in methods using shared molecular pathology [236,237,238,239] and side effect similarity [221,240].

In silico approaches have been used as complementary tools to in vivo and in vitro toxicity tests to minimize the cost, time, and animal experiments, and also to enhance the ability for toxicity prediction at the early stages of the drug development process even before a chemical is synthesized [1,2]. Such in silico techniques have been developed based on different available data resources like gene expression data [241,242], metabolomics [243], and molecular structures [244]. High-throughput technologies such as microarrays are generating a massive amount of data and huge growing data for the toxicology-related gene expression for different drugs [148]. Gene expression profile data are useful to predict potential drug-induced adverse effects at the development level of drugs [148,186,245,246,247,248,249,250]. For the development of in silico models for drug toxicity prediction, the incorporation of a wide range of computational tools is needed including databases [251,252,253,254,255], chemical descriptors of molecules [256], modeling and simulation software [256,257,258], expert systems [259,260,261], and visualization tools [262]. For instance, for the kidney, gene expression profiles have been used to train an support vector machine (SVM) predictor that could classify gene expression profiles into four categories, based on the type and severity of pathology, with high accuracy (82% sensitivity and 100% specificity) [246]. For the liver, a classifier was developed for predicting the necrosis level in tissue with accuracy as high as 90% [247]. In addition to machine learning models, network analysis approach has been utilized using gene expression profiles [248]. In another study, a network model was developed to predict chronic hepatotoxicity using sub-chronic hepatic gene expression data. Using the rat gene expression profile, the model could predict the toxic hepatopathy, hepatocellular adenoma, and changes in diffuse fatty [248]. In other research, a network model was developed to predict kidney or liver drug-induced toxicity with accuracy within 80%–97% [186]. Making the models more complicated leads to hybrid models that can be developed by taking into account the chemical descriptors in addition to gene expression data, leading to a better interpretation of the model for toxicity testing [249].

Through incorporating the gene expression data and utilizing different models (network models [248], statistical models [263], and artificial neural networks [246,247]), a number of predictors have been developed to predict toxicity effects of drugs. A majority of such studies considers only single organ toxicity. However, similar to the progress in the development of in vitro multi-organ models it is very imperative to develop in silico models predicting the drug-induced toxicity for multiple organs. In silico-based toxicological study of drug effects on multiple organs particularly the kidney and liver play essential roles in the metabolism and clearance of drugs [264]. Thus, understanding the toxic effects of drugs targeting the kidney and liver at the early stage of the drug design is very helpful [265]. Just recently, a few studies have been focused on the development of toxicity predictors for multi-organs [265,266] and more studies need to focus on multi-organs’ effects of drugs and design of appropriate predictors. Complementary works of the in silico models and in vitro multi-organs on-chip technology is expected to be a uniquely powerful tool to provide a highly predictive model in the near future for drug design.

### 4.5. Biosensors for On-Chip Technologies

Biosensors are devices that sense biological elements by the analysis of optical, electrochemical, mass, or other signals, leading to the extraction of quantitative information from the analytes [267,268]. The combination of microfluidic technology with biosensors can enhance the capabilities of microfluidic chips and extend their applications to real-time monitoring and clinical diagnostics [267]. Miniaturized biosensors provide favorable features like the low cost of reagent consumption, decreased processing time, reduced sample volume, laminar flow, parallel detection for multiple samples, and portability [267,268,269]. Optical, electrochemical, and mass-sensitive methods are the most commonly used methods for the design of such biosensors.

Optical-based biosensors use light to extract data from physical properties of a target object [269]. Using surface plasmon resonance (SPR) phenomenon, optical sensors have been developed to measure the alteration in the reflective index of a metal-coated glass substrate upon the interaction of an analyte with capture molecules [270,271,272]. Inducing light onto the metallic thin film results in an oscillation of the charge density and subsequently a drop in the light reflection at a specific light angle. In addition to SPR sensors, surface-enhanced Raman spectroscopy (SERS) has been used for molecular detection as a highly sensitive approach [273,274,275]. Using this method one can detect molecules based on their specific spectral fingerprints because different molecules generate specific spectral signals [275]. To generate an amplification on the detection signal, molecules of interest are immobilized on metal nanoparticles. The structural and chemical information about molecules can be obtained using SERS without the need for labeling [276,277,278,279,280]. However, due to the light diffraction, conventional light microscopy techniques have limited resolution and contrast. To overcome limitations, electron microscopy techniques such as scanning electron microscopy (SEM) and transmission electron microscopy (TEM) have been utilized [281]. Moreover, super-resolution methods like photo-activated localization microscopy (PALM) and structured illumination microscopy have been invented to overcome the limited-resolution challenge in conventional light microscopy techniques [282,283]. However, these techniques suffer from a high cost, complexity, and low-throughput [273]. Desired biosensors for organ-on-chip applications should be high-throughput and capable of detection of nano-sized structures. For on-chip applications, research has focused on the development of miniaturized imaging devices enabling portability and reducing cost [284,285,286,287,288]. Different approaches are under development to tackle challenges of depth-of-field (DOF), field-of-view (FOV), and resolution limitations together with making the techniques compact, simple, and cost efficient [289,290,291,292,293,294,295]. On-chip fluorescent detection [293,294,295] and in-line based lens-free integrated on-chip optical detection [289,290,291,292] have shown promising results. We have recently developed optical-based biophysical sensors specifically designed for organ-on-chip applications for real-time and long-term detection of oxygen concentrations and pH of the culture medium [296]. More advanced miniaturized and integrated optical biosensors will be developed in the near future for the monitoring of culture microenvironment for each organ of a multi-organ system.

Electrochemical biosensors in three different types of potentiometry, amperometry, and conductometry provide high sensitivity, long-term stability, reusability, and low detection limits in a simple and cost-effective manner. While electrochemical biosensors generally suffer from deterioration of stability of biological elements like enzymes, and insufficient selectivity and specificity [268,297,298], the recent advances in surface chemistry and surface functionalization have improved the performance of electrodes and made this type of biosensor very attractive in the field [268,297,298,299,300,301,302]. A fully automated electrochemical biosensor was recently integrated into a microfluidic-based bioreactor for the real-time detection of cytokines expressed by cells in an organ-on-chip platform [170]. Future electrochemical biosensors compatible with multi-organ platforms need to be able to detect multiple biomarkers simultaneously, thereby the development of multiplexed electrochemical biosensors is highly desired.

In mass-sensitive sensors, surface acoustic waves and piezoelectric effects are used for biosensing. Based on quartz resonators, several sensors have been developed including surface acoustic wave (SAW), electrochemical quartz crystal microbalance (EQCM), thickness shear mode (TSM), shear-horizontal acoustic plate mode (SH-APM), and flexural plate wave (FPW) [303,304,305,306,307,308,309,310,311].

## 5. Conclusions

Biomimetic multi-organ modeling is one of biggest challenges for the prevalent pre-clinical testing of drugs. In this review, we have provided an overview of the principles of modeling predictive multi-organ models and highlighted the application of microfluidics and tissue engineering to develop experimental multi-organ platforms for drug testing and disease modeling. In general, the platforms developed to date have been used to interconnect two or three organs together and have fairly tested a few drugs to evaluate how multi-organ systems can play significant roles in metabolisms of drugs. Examples of these multi-organs systems are intestine-liver, liver-skin, liver-kidney, and skin-liver-kidney cocultures.

While a majority of multi-organ publications have demonstrated proof-of-principle and feasibility studies, a few of these multi-organs models have been designed based on PBPK-PD rules [23,27]. Therefore, further attention is needed to (1) select a reasonable number of organs; (2) consider the scaling factors; (3) choose suitable cell types; and (4) establish an appropriate common medium circulating through multiple organs. The geometry of each organ needs to be able to convey a certain number of cells defined by PBPK-PD rules. Specific ranges of shear stress to cells need to be considered depending upon the type of cells cocultured for each organ tissue. The shear stress resulting from different perfusion and the behavior of cells under such conditions including their adherence and proliferations requires to be to be carefully assessed as a crucial design concept in the modeling of multi-organ platforms. Other challenges include the mapping of computational models with the experimental data, which requires developing predictive computational models and validating them with experimental data. Finally, there is a challenge to translate the collected data from biomimetic multi-organ models into clinically relevant therapeutics. New biosensors should be developed for organs-on-chip applications and have to be integrated into multi-organ systems in a closed-loop system to be able to monitor the tissues microenvironment and to detect cytokines and biomarkers continuously. This is particularly important for miniaturized organs given the small footprints of the system and challenges associated with sample collection methods.
